# The effects of *Cynara scolymus* and *Silybum marianum* on growth, carcass and organ characteristics, immunity, blood constitutes, liver enzymes, jejunum morphology, and fatty acid profile of breast meat in broilers

**DOI:** 10.1002/fsn3.2620

**Published:** 2021-10-06

**Authors:** Hossein Zaker‐Esteghamati, Alireza Seidavi, Mehrdad Bouyeh

**Affiliations:** ^1^ Department of Animal Science Rasht Branch, Islamic Azad University Rasht Iran

**Keywords:** antibody, chick, cynarine, diet, growth, medicinal plant, palmitic acid, silymarin, triglycerides

## Abstract

To investigate the effects of adding *Cynara scolymus* (CS) and *Silybum marianum* (SM) dry extract to the diet of broiler chickens, a 2 × 2 factorial experiment was conducted using a completely randomized design with 4 treatments and 4 replicates. Ten one‐day‐old male broiler chicks of Ross 308 strain were used in each replicate. Experimental treatments included two levels of supplemental CS and MS, 250 and 500 mg/kg of dry extract of CS and SM, which were used in combination with a basal diet for 42 days. The 42‐day trial was divided into 3 periods, 1–10 days, 11–24 days, and 25–42 days of age. The data were statistically analyzed using SAS software and means differences were tested for significance using Duncan's multiple‐range test. The results showed that the effect of the experimental treatments was not significant on growth performance in the entire period (1–42 days), carcass characteristics, and weight of immunity organs. Feed cost per kilogram of live weight increased with supplemental of CS and SM (*p* < .01). Significant effects of CS and SM supplements were observed on antibody titer against influenza virus at 28 and 42 days and also on antibody titer against sheep red blood cells (SRBC) at 35 and 42 days. Feeding 500 mg/kg of both CS and SM in the diet of broilers resulted in the highest villus height and width, crypt depth, and percentage of oleic and linolenic acids. The results of the present study suggest that feeding 250 and 500 mg/kg of dry extract of CS and SM in the diet may have positive effects on the traits studied, but to save feed costs, only250 mg/kg is recommended.

## Graphical abstract

Supplementing diets with CS and SM improved blood parameters and liver enzymes, but their effects were statistically non‐significant. Effect of application of the extracts on profile of breast fatty acids was variable, but in general, consumption of 250 mg/kg of both supplements was effective in improving profile of breast fatty acids. In general, it is recommended to supplement aflatoxin‐free diet with 250 mg/kg CS and SM to produce healthy meat, as well as to improve the quantitative and qualitative characteristics of broilers.

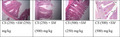

## 1 INTRODUCTION

In the last two decades, restrictions and prohibitions on the use of antibiotics in the poultry industry have led to the expanded use of antioxidants and growth‐stimulating compounds. Medicinal plants and their derivatives having antimicrobial and antioxidant compounds, as well as growth stimulants have been substituted for antibiotics in poultry diets (Brenes & Roura, 2010). Scientists have worked on improving the immune system, diet flavor, and feed conversion ratio, as well as increasing productivity while reducing the amount of cholesterol in meat and eggs. They have also investigated reducing oxidative stress, increasing feed efficiency, and improving the immune system and poultry health. Increasing production and profitability are among the characteristics that support the use of medicinal plants in poultry industry (Javandel et al., 2019; Movahhedkhah et al., 2019; Vase‐Khavari et al., 2019).

*Cynara scolymus* (CS) is a medicinal plant belonging to the chicory family. This plant is a rich source of natural antioxidants (vitamin C), flavonoids (luteolin, glycosides, cynaroside, and β‐rutinoside), carotenoids, hydrocinnamic acid, and prebiotics (fructan, inulin, and oligofructose). It is also an important source of polyphenols such as cynarine and its derivatives, which play a significant protective role in the liver. Vitamins, tannins, and organic acids are among the other effective compounds found in CS that have antimicrobial and antioxidant properties (Dranik et al., 1996; Jimene‐Escrig et al., 2003; Kaur & Gupta, 2002; Nadia et al., 2007). Zaker‐Esteghamati et al. (2020b) reported that the use of CS and its derivatives in broiler diets did not negatively affect yield, carcass characteristics, blood parameters, safety, and quality of meat; however, it lowered cholesterol and increased bird resistance to stresses, including temperature stress. These researchers reported that CS and its derivatives could be used as a useful dietary supplement in broiler diets. Various studies have reported positive effects of CS on improving the immune system, performance, feed conversion ratio, and weight gain, as well as increasing fertility, lowering cholesterol and blood lipids, protecting the liver, among others, in broilers (Edenz & Paarkhurrst, 2001; Kleessen et al., 2003; Heilmann et al., 1995; Wang et al., 2003). Stoev et al. (2000) reported that feeding 5% CS extract increased humoral immune response (increased hemagglutination inhibitory antibody titer), relative weight of organs, and on the pathomorphology, hematology, and biochemistry changes caused by ochratoxin A. Zeinab et al. (2007) showed that CS consumption had no effect on carcass characteristics except for decreasing abdominal fat. However, supplemental CS powder in the diet increased blood protein concentrations and reduced fat and blood cholesterol during the growing period.

*Silybum marianum* (SM) is a medicinal plant used in the past to treat biliary diseases and other diseases related to gastrointestinal tract. Flavonoid, resinous, and oily compounds are among the compounds that make up the active ingredients of this plant. The most important flavonoids in SM fruit are silybin, silychristin, and silydianin, which are collectively called silymarin. Dried seed extract of SM contains 1%–4% of silymarin, which includes flavonoids of silybin A and B, silydianin, silychristin, and dehydro‐silybin (Omidbiagi, 2005; Schulz et al., 1997). Silymarin has antioxidant and anti‐inflammatory properties and reduces blood cholesterol and fat. It was also observed to improve immune response and reduce the risk of atherosclerosis (Gupta et al., 2000; Horvath et al., 2001).

Consumption of SM and its derivatives in aflatoxin‐contaminated diets improved health and yield of broilers (Zaker‐Esteghamati et al., 2020a). Results of various studies have shown that silymarin improves performance, carcass percentage, and lowers cholesterol, protects the liver against liver cirrhosis, and improves the immune system in broilers (Chand et al., 2011; Tedesco et al., 2004). Schiavone et al. (2007) reported that silymarin from the extract of SM fruit did not affect growth of Ross 308 strain broiler chickens, but reduced fat in breasts and thighs and increased muscle resistance to oxidative stress. Silymarin supplementation also improved meat quality and reduced mortality. Alhidary et al. (2017) suggested that the anti‐aflatoxin activity of SM protects the liver in poultry. They reported that the active ingredients in SM reduced the harmful effects of aflatoxin and thus increased productivity and health of broilers. Another study found that adding SM seeds to the diet had no effect on live weight gain and feed conversion ratio. However, supplemental SM seeds lowered cholesterol and activity of the enzymes alanine aminotransferase (ALT) and aspartate aminotransferase (AST). Supplementation of SM reduced lipid content and increased glycogen concentrations in the liver (Suchy et al., 2008).

In previous studies, powders and effective compounds of SM and CS were used mainly in diets contaminated with aflatoxins and few studies examined the combined effect of dry extracts of these plants. There is also no comprehensive research on the effects of CS and SM, fed individually or together, on production parameters of broilers under normal conditions.

The choleretic and cholagogue effect of natural extracts has been associated with improved fat digestion (Abdel‐Salam et al., 2012). There are no reports that have investigated the effect of CS and SM extracts on choleretic and cholagogue effects in broilers. There is some evidence for synergistic effects of CS and SM in birds. Therefore, the aim of this study was to investigate the effect of two levels of dry extract of CS and SM supplemented in a diet free of aflatoxin, on growth performance, carcass characteristics and organs of the gastrointestinal tract, blood parameters, immune system, small intestinal morphology, liver enzymes, and fatty acid profiles of breast meat in broiler chickens.

## 2 MATERIALS AND METHODS

The use and care of birds in this study were approved by the Rasht Branch, Islamic Azad University from the point of Ethical issues (971218). All the experimental procedures described herein were also approved by the Rasht Branch, Islamic Azad University. Care was taken to minimize the number of birds used.

The study was conducted at a private broiler farm in Masal, Iran. The experiment lasted for 42 days and was conducted using 160 one‐day‐old male chicks of commercial Ross 308 strain. Average weight of chicks at the start of the study was 45 ± 2 g. The experimental design was a 2 × 2 factorial with 4 treatments and 4 replicates of 10 chicks each. All chicks were fed a basal diet (Table 1) supplemented with two concentrations of dry extracts of CS and SM. The four experimental treatments were as follows:

1. 250 mg/kg of each of CS and SM.
2. 250 mg/kg of *CS* +500 mg/kg of SM.
3. 500 mg/kg of *CS* +250 mg/kg of SM.
4. 500 mg/kg of each of CS and SM.

**TABLE 1:** Ingredient and calculated chemical composition of three experimental diets fed from 1 to 42 days of age

	Starter diet	Grower diet	Finisher diet
	(1–10 days)	(11–24 days)	(25–42 days)
Ingredients (% as‐fed)			
Corn	47.03	59.60	65.99
Wheat	5.58	5.00	5.00
Soybean meal (44% Crude protein)	29.02	16.15	10.28
Corn gluten	10.00	11.48	11.50
Soy oil	3.50	3.40	3.09
Limestone	1.45	1.23	1.00
Di‐calcium phosphate	1.95	1.80	1.83
Salt	0.20	0.20	0.20
Vitamin and mineral^a^ supplements^a^	0.50	0.50	0.50
DL‐methionine	0.52	0.58	0.57
L‐lysine hydrochloride	0.25	0.06	0.04
Chemical composition			
Metabolizable energy (kcal/kg)	2,950	3,000	3,050
Crude protein (%)	22	20	19
Lysine (%)	1.3	1.2	1.1
Methionine (%)	0.56	0.54	0.52
Met+Cys (%)	0.92	0.90	0.88
Calcium (%)	1.04	0.95	0.92
Available phosphorus	0.52	0.47	0.41

^a^ The amount of vitamins and minerals per kg of the final diet were: vitamin A, 9,000 IU; vitamin D3, 3,000 IU; vitamin E, 18 IU; vitamin K3, 3 mg; vitamin B1(thiamine), 1/8 mg; vitamin B2 (riboflavin),6 mg; vitamin B6 (pyridoxine), 3 mg; vitamin B12 (cyanocobalamin), 0/012 mg; vitamin B3 (niacin), 30 mg; vitamin B9 (folic acid), 1 mg; vitamin H3 (Biotin), 0/24 mg; vitamin B5 (pantothenic acid), 10 mg; choline, 100 mg; Mn, 100 mg; zinc, 80 mg; iron, 10 mg; Cu, 1 mg; I, 0/2 mg.

The two dry extracts were purchased from Barij Essence Pharmaceutical Company (Kashan, Iran). The diets were ground and adjusted to the nutritional needs of chicks during the starter (1–10 days), grower (11–24 days), and finisher (25–42 days) periods. Diets were formulated to meet the minimum nutrients recommended for the Ross 308 strain (Manual, 2012). During the experiment, all chicks had free access to water and feed. Environmental conditions of the breeding hall were standardized for all groups at 23 hr of exposure to light, 60%–75% relative humidity and 32°C. As the chicks grew older, the temperature in the breeding hall was reduced by 3°C per week until the temperature reached 23°C. All birds were vaccinated against infectious bronchitis (day 10), Newcastle disease (days 4, 21, and 35), and infectious Bursal disease (day 12) (NDV; strain Viscerotropicvelogenic). All vaccines were obtained from Razi Serum and Vaccine Institute (Karaj, Iran).

### 2.1 Sample and data collection

#### 2.1.1 Growth performance and economic efficiency

At the end of each period (starter, grower, and finisher), weight gain of chicks and the amount of feed in each feeding container were recorded using a digital scale (A&D GF‐300‐Japan) with an accuracy of 0.001 g. Feed intake was calculated by deducting the remaining amount of feed in each feeding container from the amount at beginning of each period. The feed conversion ratio was calculated by dividing the amount of feed intake by weight gain of chicks during the starter, grower, and finisher periods (Sigolo et al., 2019). The following formula was used to calculate the European production factor (Hajati et al., 2015):

European production index =mean live weight (g) × durability percentage/feed conversion ratio×number of breeding days ×100.

The daily price of dry extracts was calculated separately for each treatment and the cost of feed (in Rials) consumed per kilogram of live chicks was calculated during the 42 days of the trial.

#### 2.1.2 Carcass characteristics and digestive organs

At the end of the experiment, birds were deprived of food for 2 hr. Two birds from each replicate with similar average weight were slaughtered and weighed. Weight of various carcass components and weight of digestive organs (pancreas, liver, gizzard, heart, ventricular fat, duodenum, jejunum, colon, cecum, and ileum) were recorded (Shabani et al., 2015).

#### 2.1.3 Blood metabolites and digestive enzymes

Two birds from each treatment with average weight were picked at random, and 5 ml of blood was collected from the wing veins. Blood samples were mixed and held at room temperature for 12 hr, then centrifuged at 5,000 rpm for 3 min (Eppendorf 5702, Germany), and serum collected and stored at 20°C until analyses. Serum was thawed at room temperature and concentrations of glucose, triglyceride, cholesterol, total protein, albumin, globulin, very low‐density lipoprotein (VLDL), high‐density lipoprotein (HDL), low‐density lipoprotein (LDL), ATL, alkaline phosphatase, creatine kinase, and lactate dehydrogenase were measured using commercial kits (Pars Azmoon) and by auto analyzer (Hitachi 917, Japan) based on the methods of Golrokh et al. (2016).

#### 2.1.4 Immune response

To investigate humoral immunity, the immunization of broiler chickens against SRBC was performed according to Lerner et al. (1971). On days 28 and 36, 0.1cc SRBC was injected into the wing vein of two birds from each pen. On days 35 and 42, blood samples of selected chicks were collected and antibody level against SRBC was measured by the hemagglutination method (Seidavi et al., 2014). Microhemagglutination V plates were used to measure antibody titers.

The Van derzipp method was used to measure total antibodies. To measure total anti‐SRBC, 50 μl of serum was mixed with 50 μl of phosphate‐buffered saline (PBS) inside a micro titer plate and serial dilutions from 1.2 to 1.256 were prepared. In the next step, 50 μl of 2% SRBC suspension were added to each well and maintained at room temperature for 4 to 5 hr. Titers were based on Log 2 of the highest dilution, which was showed complete agglutination (Pourhossein et al., 2015).

To evaluate Newcastle (NDV) and influenza (AIV) titers on days 28 and 42 of experiment, two additional chicks were selected from each pen and blood samples were collected from their wing veins. A hemagglutination inhibition (HI) assay based on the World Organization of Animal Health standard was used to evaluate serum levels of NDV and AIV. First, 25 μl of PBS were added into each well, then 25 μl of serum was pipetted into the first well, and their dilution was performed until the last well. Then, 25 µl of Newcastle and influenza antigens were added to all wells and the microplate was placed on a mechanical shaker for 1 min and stored at 25˚C for 30 min. In the following step, 25 microliters of 1% red blood cells were added to all wells and the microplate was placed on a mechanical shaker for 15 s. After shaking was complete, microplates were incubated at 25˚C for 30 min before recording the results. A 4‐unit antigen (Pasouk, Iran) was used to perform the Hemagglutination‐inhibition (HI) test. Dilution titers were performed based on a log 2 scale. Red blood cells (1%) were obtained from SPF chicks. On day 42, blood from two birds from each pen was collected, mixed to form a composite, and then transferred to tubes containing anticoagulants to count the total number of white blood cells and their differential counts. Determination of blood cells was performed by staining, cell differentiation, and counting under light microscopy (Seidavi et al., 2014). At the end of the experiment, the birds were deprived of feed for 2 hr and two birds from each replicate were slaughtered and weight of spleen, bursa of Fabricius and thymus measured (Shabani et al., 2015).

### 2.2 Morphology of jejunum tissue

Of the remaining birds, two from each treatment were slaughtered and the middle part of the jejunum was collected and immediately transferred into plastic containers containing 10% formalin to prevent tissue degradation and to maintain their physical structure. The samples were later dehydrated using a tissue processor (1512, ‐Lab SC) for 12 hr. Slices of jejunum tissue, 5 μm diameter, were stained using hematoxylin–eosin and lamelation. Villus length, crypt depth, and muscle thickness were measured using a microscope (CX23 Olympus, Japan), and villus height to crypt depth ratio was calculated (Sakamoto et al., 2000).

### 2.3 Fatty acid profile in breast meat

One bird from each treatment was slaughtered to measure the fatty acids at 42 days of age. A sample of breast meat from each bird was transferred to laboratory, where 20 g of minced breast meat were mixed with 50 ml of methanol for 30 min before adding 40 ml of hexane and stirring for 20 min. The mixing operation lasted until two layers were formed. The top layer containing the methyl ester, and lipid was analyzed for individual fatty acids by gas chromatographic (GC‐MS model, USA) as described by (Folch et al., 1957).

### 2.4 Statistical analysis

All data were analyzed using SAS statistical software (SAS Institute, 2002). Mean comparisons were performed using Duncan's multiple‐range test at 5% probability level. The design of this study was a complete randomized 2 × 2 factorial, and the statistical model applied was:

$$Y_{\text{ijk}}=\mu +A_{i}+B_{j}+AB_{\text{ij}}+E_{\text{ijk}},$$

where *Y*_ijk_ is observation value; µ is overall mean; *A*_i_ is effect of CS in the diet (250 or 500 mg/kg); *B*_j_ is effect of SM in the diet (250 or 500 mg/kg); *AB*_ij_ is interaction effect of supplements; *E*_ijk_ is experimental error.

## 3 RESULTS AND DISCUSSION

### 3.1 Growth performance

Feed intake in the starter (1–10 days) and grower (11–24 days) periods was higher (*p* <.05) 250 mg/kg CS for whereas feed conversion ratio in the finishing (25–42 days) period was highest for 500 mg/days of CS. Feed intake increased (*p* <.05) with 500 mg/kg SM only during the starter period. The highest feed intake was observed when 250 mg/kg CS and 500 mg/kg SM were fed during the starter (19.28 g/chick/day) and grower (81.39 g/chick/day) periods compared with all other treatments (Table 2). Feed cost per kilogram of live weight were lowest (*p* <.05) when 250 mg/kg of both CS and MS were added to the diet (53,527.70 Rial/kg) and highest when 500 mg/kg of both CS and MS supplements were added (59,600.20 Rial/kg) (Table 3).

**TABLE 2:** Feed intake and growth of Ross 308 broilers fed diets containing two concentration (250 or 500 mg/kg) of *Cynara scolymus* (CS) and *Silybum marianum* (SM) during starter (1–10 days), grower (11–24 days), finisher (25–42 days), and phases and whole period (1–42 days)

		1–10 days of age			11–24 days of age			25–42 days of age			1–42 days of age		
		Feed intake (g/chick/day)	Weight gain (g/chick/ day)	Feed conversion ratio	Feed intake (g/chick/ day)	Weight gain (g/chick/ day)	Feed conversion ratio	Feed intake (g/chick/ day)	Weight gain (g/chick/ day)	Feed conversion ratio	Feed intake (g/chick/ day)	Weight gain (g/chick/ day)	Feed conversion ratio
CS (mg/kg)	250	18.04^a^	11.30	1.61	79.48^a^	33.11	2.42	151.86	95.63	1.60^b^	95.87	54.71	1.76
	500	16.22^b^	10.48	1.57	60.48^b^	29.41	2.07	159.15	94.77	1.68^a^	92.24	52.92	1.75
SM (mg/kg)	250	16.15^b^	10.88	1.50	67.82	30.50	2.22	155.63	95.50	1.64	93.15	53.68	1.74
	500	18.11^a^	10.90	1.67	72.14	32.02	2.27	155.39	94.90	1.64	94.96	53.94	1.76
CS (250) × SM (250)		16.80^b^	11.35	1.49	77.57^ab^	32.97	2.37	153.56	96.72	1.60	95.67	55.14	1.74
CS (250) × SM (500)		19.28^a^	11.25	1.72	81.39^a^	33.24	2.46	150.16	94.53	1.59	96.07	54.27	1.77
CS (500) ×SM (250)		15.50^b^	10.40	1.51	58.07^c^	28.03	2.06	157.69	94.27	1.67	90.63	52.22	1.74
CS (500) ×SM (500)		16.95^b^	10.55	1.62	62.89^bc^	30.79	2.08	160.61	95.27	1.69	93.84	53.61	1.75
*SEM*		0.66	0.66	0.08	4.89	1.69	0.17	7.34	4.85	0.04	3.28	2.05	0.01

Note

^a,b,c^Means within each column of dietary treatments with different letters are statistically different (*p* <.05). *SEM*: Standard Error of Means

**TABLE 3:** Economic performance of Ross 3,008 broilers at 42 days of age fed diets containing two concentrations (250 or 500 mg/kg) of *Cynara scolymus* (CS) and *Silybum marianum* (SM)

		Average weight of chick (g)	Feed cost per kg live weight (Rial/kg)	European production index
CS (mg/kg)	250	2,337.80	55,235.8^b^	317.74
	500	2,262.40	58,153.45^a^	309.24
SM (mg/kg)	250	2,294.80	55,117.2^b^	315.02
	500	2,305.40	58,272.05^a^	311.97
CS(250) ×SM (250)		2,356.3	53,527.7^c^	323.57
CS (250) × SM (500)		2,319.3	56,943.9^b^	311.91
CS (500) ×SM (250)		2,233.3	56,706.7^b^	306.46
CS (500) ×SM (500)		2,291.5	59,600.2^a^	312.02
*SEM*		86.28	45.21	13.2

Note

^a,b,c^Means within each column of dietary treatments with different letters are statistically different (*p* <.05). *SEM*: Standard Error of Means.

Researchers have reported that cynarine in CS improves the performance of broilers by increasing the digestibility of lipids and improves absorption of amino acids (Edenz & Paarkhurrst, 2001; Heilmann et al., 1995). Tajodini et al. (2015) did not observe any effect of supplemental CS on weight gain, feed conversion ratio, or feed intake of broiler chickens, which is consistent with results of the present study. Zeinab et al. (2007) stated that supplemental CS in broiler diets increased feed cost, but adding 4% CS extract to the basal diet improved the economic performance. In the present study, supplementation of 250 mg/kg of both CS and SM had the lowest feed cost per kilogram of live weight, the heaviest weight at 42 days, and the highest European production factor.

Supplementing poultry diets with SM increases resistance to stress by reducing harmful microbial populations of the gut and improving the health of the gastrointestinal tract. It also increases nutrient intake and performance and improves feed conversion ratio (Chand et al., 2011; Rashidi et al., 2015; Tedesco et al., 2004; Windisch et al., 2008). Gharahveysi (2017) stated that the inclusion of SM plant powder (0%, 0.3%, and 3%) in diet of Ross 308 strain broilers reduced average feed intake and average body weight throughout the breeding period. Suchy et al. (2008) reported that adding 0.2% or 1% milk thistle (SM) seed tablets in the diet of Ross 308 strain broilers had no effect on functional traits. Similar results were reported by Schiavone et al. (2007) who reported that dry extract of SM fruit (silymarin) had no effect on growth performance of broiler, which is consistent with results of the present study.

### 3.2 Carcass characteristics and digestive organs

Different concentrations of CS and SM did not affect carcass characteristics or weight of digestive organs except on for the relative weight of proventriculus, which was higher (*p* <.05) for broilers fed CS and MS at 250 or 500 mg/kg compared with the other treatment combinations (Tables 4 and 5).

**TABLE 4:** Means of carcass traits of Ross 308 broilers at 42 days of age fed diets containing two concentrations (250 or 500 mg/kg) of Cynara scolymus (CS) and Silybum marianum (SM)

		Live body weight (g)	Defeat her body (g)	Full abdomen carcass weight (g)	Empty abdomen carcass weight (g)	Eviscerated carcass (%)	Relative weight of crop (%)	Relative weight of breast (%)	Relative weight of drumsticks (thighs) (%)	Relative weight of wings (%)	Relative weight of abdominal fat (%)	Relative weight of pancreas (%)
CS (mg/kg)	250	2,696.25	2,492.15	2,325.90	2040.40	81.82	0.47	32.47	28.14	6.40	0.67	0.23
	500	2,709.25	2,518.55	2,354.80	2072.65	82.30	0.43	32.40	28.83	5.92	0.51	0.20
SM (mg/kg)	250	2,675.50	2,483.55	2,321.05	2038.15	82.01	0.44	32.16	28.61	6.15	0.65	0.22
	500	2,730.00	2,527.15	2,359.65	2074.90	82.11	0.46	32.71	28.36	6.16	0.53	0.21
CS(250) ×SM (250)		2,627.50	2,428.80	2,263.80	1970.30	81.02	0.5	31.62	27.59^b^	6.43	0.74	0.26^a^
CS (250) × SM (500)		2,765.00	2,555.50	2,388.00	2,110.50	82.61	0.43	33.31	28.69^ab^	6.36	0.59	0.20^ab^
CS (500) ×SM (250)		2,723.50	2,538.30	2,378.30	2,106.00	83.00	0.37	32.70	29.62^a^	5.87	0.56	0.18^b^
CS (500) ×SM (500)		2,695.00	2,498.80	2,331.30	2039.30	81.60	0.48	32.10	28.03^b^	5.96	0.46	0.22^ab^
*SEM*		114	108	103	91	0.61	0.04	0.87	0.48	0.33	0.11	0.02

Note

None of the differences between means are statistically different (*p* >.05). *SEM*: Standard Error of Means

**TABLE 5:** Relative weight of organs as percentage of body weight in Ross 308 broilers at 42 days of age fed diets containing two concentrations (250 or 500 mg/kg) of *Cynara scolymus* (CS) and *Silybum marianum* (SM)

		Gizzard (Ventriculus) (%)	Heart (%)	Liver (%)	Proventriculus (%)
CS (mg/kg)	250	2.22	0.56	2.19	0.39
	500	2.23	0.52	2.24	0.41
SM (mg/kg)	250	2.33	0.56	2.13	0.40
	500	2.12	0.52	2.30	0.40
CS(250) ×SM (250)		2.58^a^	0.59^a^	2.25	0.43^ab^
CS (250) × SM (500)		1.86^b^	0.53^ab^	2.12	0.35^b^
CS (500) ×SM (250)		2.07^ab^	0.52^b^	2.01	0.37^b^
CS (500) ×SM (500)		2.38^ab^	0.51^b^	2.47	0.45^a^
*SEM*		0.18	0.02	0.16	0.02

Note

^a,b^Means within each column of dietary treatments with different letters are statistically different (*p* <.05). *SEM*: Standard Error of Means

Boroumandnia et al. (2014) observed that supplemental CS extract (0, 100, 300, and 500 mg/L) had no effect on body weight, carcass weight, carcass yield, thigh weight, breast weight, gizzard weight, liver weight, or intestine weight. Stastnik et al. (2016) reported that supplementing 5% or 15% SM seed tablets in the diet of Ross 308 strain broilers did not affect carcass characteristics. Rashidi et al. (2015) stated that adding SM seeds (0.5, 1, 1.5, or 2%) in the diet of broilers had no effect on weights of carcass, breast, thigh, and ventricular fat. Afshin et al. (2017) did not observe any effect of supplementing 0.5% seed powder, 1% plant powder, or 600 or 1,000 ppm of SM extract on carcass characteristics of broilers. The results of our current present study are in agreement with previous research cited. Due to the antimicrobial and antioxidant properties of CS and SM extracts, as well as their effect on enhance diet flavor, it can be inferred that feeding these extracts may be beneficial through the reduction harmful microbes in gastrointestinal tract and reducing stress, which improves nutrient absorption. This supports improved digestion system function, general health, and carcass characteristics. Raberfroid (2005) reported that the active ingredients in CS extracts, such as inulin and oligofructoses, reduce triglyceride production and fat accumulation in the body. Newman et al. (2002) suggested that the use of unsaturated fatty acids in diet reduces accumulation of ventricular fat and total body fat. Khan et al. (2007) reported that using SM in diet could be effective in reducing ventricular fat accumulation due to its high content of unsaturated fats.

### 3.3 Intestine weight and morphology

No differences were observed for feeding combinations of CS and SM on the relative weight of rectum, duodenum, jejunum, colon, right cecum, and left cecum; however, broilers fed 500 mg/kg of CS had greater (*p* <.05) ileum weight (Table 6). Supplemental SM to broilers was reported to increase both the length and weight of intestine (Kalantar et al., 2014). Presence of fiber in diet is one of the reasons cited for the increase in intestinal weight. Diets containing SM had high more fiber. Intestinal movement and activity increase during digestion and absorption of fibers resulting in increased length and weight (Rashidi et al., 2015; Rezaei et al., 2011). The CS has prebiotic properties due to their fructans (Nadia et al., 2007). Therefore, it is assumed that CS improves intestinal characteristics by stimulating production of probiotics to improve nutrient digestion and absorption.

**TABLE 6:** Relative weight of intestinal segments as a percentage of body weight of Ross 308 broilers at 42 days of age fed diets containing two concentrations (250 or 500 mg/kg) of *Cynara scolymus* (CS) and *Silybum marianum* (SM)

	Rectum (%)	Duodenum (%)	Jejunum (%)	Ileum (%)	Colon (%)	Right cecum (%)	Right cecum (%)	Left cecum (%)
CS (mg/kg)	250	0.25	0.57	1.39	0.49^a^	0.46	0.23	0.23
	500	0.24	0.60	1.33	0.55^b^	0.42	0.21	0.21
SM (mg/kg)	250	0.23	0.59	1.39	0.52	0.46	0.23	0.23
	500	0.26	0.58	1.34	0.52	0.42	0.21	0.21
CS(250) ×SM (250)		0.23	0.59	1.51	0.50	0.50	0.25	0.25
CS (250) × SM (500)		0.27	0.55	1.27	0.48	0.42	0.21	0.21
CS (500) ×SM (250)		0.22	0.59	1.26	0.54	0.41	0.21	0.20
CS (500) ×SM (500)		0.25	0.60	1.40	0.55	0.42	0.21	0.21
*SEM*		0.02	0.02	0.09	0.03	0.03	0.01	0.02

Note

^a,b^Means within each column of dietary treatments with different letters are statistically different (*p* <.05). *SEM*: Standard Error of Means.

### 3.4 Blood parameters and digestive enzymes

Supplemental CS and SM did not alter the biochemical parameters of blood when fed to growing broilers (Table 7). No differences were observed in concentrations of alkaline phosphatase, alanine transaminase, lactate dehydrogenase, and creatine kinase (Table 8).

**TABLE 7:** Blood metabolite concentrations of Ross 308 broilers at 42 days of age fed diets containing two concentrations (250 or 500 mg/kg) of *Cynara scolymus* (CS) and *Silybum marianum* (SM)

		Cholesterol (mg/dl)	Triglycerides (mg/dl)	VLDL (mg/dl)	HDL (mg/dl)	LDL (mg/dl)	LDL/HDL	Glucose (mg/dl)	Total protein (g/dl)	Albumin (g/dl)	Globulin (g/dl)
CS (mg/kg)	250	143.25	117.25	23.45	77.25	37.38	0.49	177.75	3.96	1.90	2.06
	500	131.13	82.25	16.45	73.50	37.25	0.51	193.25	3.44	1.93	1.52
SM (mg/kg)	250	133.00	97.25	19.45	73.75	35.75	0.49	178.00	3.88	1.99	1.89
	500	141.38	102.25	20.45	77.00	38.88	0.51	193.00	3.52	1.84	1.68
CS (250) ×SM (250)		139.50	126.50	25.30	75.75	36.50	0.48	173.50	4.33	2.00	2.33
CS (250) × SM (500)		147.00	108.00	21.60	78.75	38.25	0.49	182.00	3.58	1.80	1.78
CS (500) ×SM (250)		126.50	68.00	13.60	71.75	35.00	0.49	182.50	3.43	1.98	1.45
CS (500) ×SM (500)		135.75	96.50	19.30	75.25	39.50	0.52	204.00	3.45	1.88	1.58
*SEM*		9.55	25.88	5.18	3.64	4.37	0.04	11.26	0.49	0.39	0.29

Note

VLDL (very low‐density lipoprotein), HDL Cholesterol (high‐density lipoproteins), LDL cholesterol (low‐density lipoproteins).

None of the differences between means were different (*p* >.05), *SEM*: Standard Error of Means.

**TABLE 8:** Liver enzyme concentrations at 42 days of age in Ross 308 broilers fed diets containing two concentrations (250 or 500 mg/kg) of *Cynara scolymus* (CS) and *Silybum marianum* (SM)

		Alkaline phosphatase (U/L)	Alanine transaminase (IU/L)	Lactate dehydrogenase (IU/L)	Creatine kinase (IU/L)
CS (mg/kg)	250	3,866.5	357.05	5,305	25,350
	500	3,877	372.8	5,738	23,327
SM (mg/kg)	250	3,790.5	354.3	5,475.5	21,365
	500	3,953	375.55	5,567.5	27,312
CS (250) ×SM (250)		4,095	278.3	5,150	12,448
CS (250) × SM (500)		3,638	435.8	5,460	38,252
CS (500) ×SM (250)		3,486	430.3	5,801	30,282
CS (500) ×SM (500)		4,268	315.3	5,675	16,372
*SEM*		717.49	90.7	1,244	11,533

Note

No differences were observed among treatments (*p* >.05), *SEM*: Standard Error of Means.

Ghohari et al. (2013) reported that supplemental CS in broiler diets had no effect on blood parameters, which is consistent with results of the present study. Silymarin (an active ingredient in SM) is a powerful antioxidant that has been shown to improve blood parameters and liver metabolism (Rashidi et al., 2015; Sobolova et al., 2006). No differences were observed among the experimental treatments on immune parameters and (Table 10), the combination of 500 mg/kg CS plus 250 mg/kg SM numerically reduced cholesterol, triglycerides, VLDL, and LDL compared with the other treatments (Table 7). The treatment in which 250 mg/kg of both supplements were added was the most successful in increasing total protein, albumin, and globulin.

Afshin et al. (2017) did not observe any differences in cholesterol, high‐density lipoprotein, low‐density lipoprotein, or lactate dehydrogenase for broiler fed a diet contaminated with aflatoxin and supplemented with seed powder, full plant powder, or SM extract. However, ALT concentration with the use of seed powder, full plant powder, and extract of SM compared with normal concentrations. The decrease in ALT concentrations with supplementation of various forms of SM plant in an aflatoxin‐contaminated diet indicates a decrease in the toxic effect of aflatoxin on liver cells by the active ingredients of this medicinal plant. Previous studies have reported no effect of supplemental SM on blood metabolites (FaniMakki et al., 2013; Sourani et al., 2016) and concentrations of serum albumin and globulin of broilers (Kamali & SeyedMostafaei, 2014), which is consistent with results of the present study. Tedesco et al. (2004) reported that adding 600 ppm silymarin to a diet contaminated with 0.08 mg/kg aflatoxin B1 increased the ALT activity. Effects of the treatments in this study on other biochemical blood properties, including protein, albumin, globulin, blood urea nitrogen, glucose, total bilirubin, direct bilirubin, indirect bilirubin, AST, ɣ‐glutamyl transferase, calcium, and phosphorus, were not significant.

Silymarin appears to inhibit absorption of toxins by binding to membrane receptors in liver cells altering the structure of membrane receptor. On the contrary, the antioxidant properties of SM prevent metabolic disorders of these cells by inhibiting lipid peroxidation in liver cells. Silymarin has also been reported to have a positive effect on preventing altered lipoprotein metabolism, which is a major cause of cellular and metabolic problems in patients with hepatic impairment (Halim et al., 1997; Schonfeld et al., 1997). The effect of different concentrations of SM on the reduction in hepatic enzymes in the blood (Gharahveysi, 2018) and lack of difference in hepatic enzyme activity (FaniMakki et al., 2013; Gharahveysi, 2017) have previously been reported, which is consistent with results of our present study. It should be noted that although no differences were observed among the experimental treatments on liver enzyme activity in the present study, the lowest activity of ALT, lactate dehydrogenase, and creatine kinase enzyme was recorded with the lower dose (250 mg/kg) of the two supplements suggesting a protective effect of that treatment on liver health.

### 3.5 Immune system

No differences were observed among treatments on percentage of relative weights of the immune organs (thymus, spleen, and bursa of Fabricius, Table 9). Supplemental CS and SM did not alter white blood cell count, neutrophils, lymphocytes, eosinophils, and antibody titers against Newcastle disease at 28 and 42 days of age (Table 10). However, antibody titer against influenza virus at 28 days was higher (*p* <.05) when CS was supplemented at 500 mg/day compared with 250 mg/day. An interaction was observed at 42 day for antibody titer against influenza and was highest when 500 mg/day of both CS and MS, intermediate for 500 mg/kg CS plus 250 mg/kg MS, and lowest for 250 mg/kg CS plus 500 mg/kg MS. Antibody titers were highest (*p* <.05) for 250 mg/kg CS plus 250 mg/kg MS at 35 days compared with the other treatments.

**TABLE 9:** Immunity related organs as a percentage of body weight at 42 days of age in Ross 308 broilers fed diets containing two concentrations (250 or 500 mg/day) of *Cynara scolymus* (CS) and *Silybum marianum* (SM)

		Thymus (%)	Spleen (%)	Bursa of Fabricius (%)
CS (mg/kg)	250	0.39	0.11	0.10
	500	0.39	0.12	0.08
SM (mg/kg)	250	0.39	0.11	0.1
	500	0.39	0.11	0.08
CS(250) ×SM (250)		0.38	0.1	0.11
CS (250) × SM (500)		0.4	0.11	0.09
CS (500) ×SM (250)		0.4	0.12	0.08
CS (500) ×SM (500)		0.37	0.11	0.07
*SEM*		0.02	0.01	0.01

Note

No differences were observed among treatments (*p* >.05), *SEM*: Standard Error of Means.

**TABLE 10:** Immune response of Ross 308 broilers fed diets containing two concentrations (250 or 500 mg/kg) of *Cynara scolymus* (CS) and *Silybum marianum* (SM)

		White blood cells (*n* × 103/ml)	Neutrophils (%)	Lymphocytes (%)	Eosinophils (%)	Newcastle disease (log 2)		Avian influenza (log 2)		Sheep red blood cell	
						28 days	42 days	28 days	42 days	35 days	42 days
CS (mg/kg)	250	2,600	45.50	49.75	4.75	2.63	6.13	1.50^b^	4.38^b^	3.38	6.38
	500	1,500	42.63	52.75	4.63	2.63	6.75	2.50^a^	6.00^a^	2.50	6.38
SM (mg/kg)	250	2,350	44.88	49.00	6.13	2.25	6.25	2.00	5.13	3.50	6.50
	500	1,750	43.25	53.5	3.25	3.00	6.63	2.00	5.25	2.38	6.25
CS (250) ×SM (250)		3,300	44.75	50.00	5.25	2.00	6.25	1.50^b^	4.75^bc^	4.75^a^	7.25
CS (250) × SM (500)		1900	46.25	49.50	4.25	3.25	6.00	1.50^b^	4.00^c^	2.00^b^	5.5
CS (500) ×SM (250)		1,400	45.00	48.00	7.00	2.50	6.25	2.5^a^	5.50^ab^	2.25^b^	5.75
CS (500) ×SM (500)		1,600	40.25	57.50	2.25	2.75	7.25	2.5^a^	6.50^a^	2.75^b^	7.00
*SEM*		890	6.75	6.62	1.54	0.77	0.71	0.29	0.43	0.54	0.59

Note

^a,b,c^Means within each column of dietary treatments with different letters are statistically different (*p* <.05). *SEM*: Standard Error of Means.

Improvement in immune parameters of broiler chickens grown under heat stress conditions has been reported when supplemental CS powder was fed (Effati et al., 2014). Stoev et al. (2000) reported that feeding 5% CS extract had a protective effect on humoral immune response (increased hemagglutination inhibitory antibody titer), relative weight of organs, and on changes in pathomorphology, hematology, and biochemistry caused by acratoxin A.

Research has shown that the use of different concentrations of SM seed powder (0%, 0.2%, 0.4%, and 0.6%) in broiler diets improves the quantity of antibodies against SRBC at 42 days (Jafari et al., 2016). Silymarin has antioxidant properties which protect tissues from oxidative stress and damage caused by free radicals. In addition, antioxidants prevent peroxidation of membrane of immune cells preventing the weakening of the immune system by maintaining fluidity of cell membranes (Bendich, 1993). There have been reports that plant flavonoid compounds, including silymarin in SM, can boost the immune system and increase antibody production in animals due to its antibacterial properties and vitamin C enhancing activity (Cook & Samman, 1996). Examination of humoral immunity parameters of Ross 308 strain broiler chickens fed a diet supplemented with different levels of raw and heated SM seeds indicated that SRBC concentrations increased with SM supplementation compared with the control (Mozaffarpour Toubkanlou et al., 2017). Amiri Dumari et al. (2014) reported that feeding SM (0.5 and 1%) had no effect on antibody titers of Newcastle and influenza, but Mushtaq et al. (2013) reported that supplementation of SM extract and barberry in broiler diets increased antibody quantity against Newcastle and bronchitis.

### 3.6 Morphological characteristics of jejunum

**FIGURE 1:**
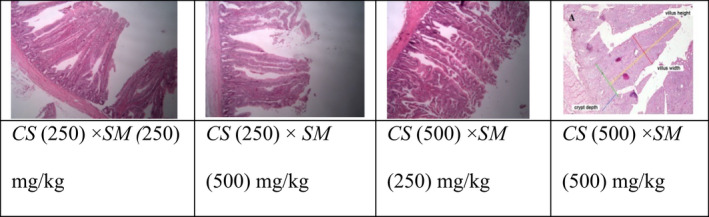
Morphological image of jejunum on day 42 in Ross 308 broilers fed diets supplemented with two concentrations (250 or 500 mg/kg) of *Cynara scolymus* (CS) and *Silybum marianum* (SM)

The morphological characteristics of the jejunum are presented in Table 11 and Figure 1. Villus height was numerically higher with increasing concentrations of CS and SM, and the shortest villus was observed when 250 mg/kg of both dry extracts were fed (956.25 µm), while the highest occurred with the high doses (500 mg/kg) (1,205.08 µm). The latter treatment also resulted in the maximum villus width (206.71µm) and the maximum crypt depth (179.88 µm). Thickness of muscle layer and villus height to crypt depth ratio were highest in the treatment with 500 mg/kg CS and 250 mg/kg SM and the lowest in the treatment with 250 mg/kg of both supplements. In a study by Hasheminejad et al. (2015), supplementation of two concentrations of SM seeds (5 and 10 g/kg) in broiler diets did not affect villus height or length, crypt depth, goblet cell number, and ratio of villus height to crypt depth.

**TABLE 11:** Morphological indices of jejunum on day 42 in Ross 308 broilers fed diets containing two concentrations (250 or 500 mg/kg) of *Cynara scolymus* (CS) and *Silybum marianum* (SM)

		Villus height (µm)	Villus width (µm)	Crypt depth (µm)	Muscle layer thickness (µm)	Villus height: crypt depth ratio (%)
CS (mg/kg)	250	1,029.50	188.70	165.75	124.45	6.38
	500	1,151.00	201.45	168.85	179.45	6.96
SM (mg/kg)	250	1,026.50	197.95	156.00	145.35	6.69
	500	1,154.00	192.20	178.60	158.55	6.64
CS (250) ×SM (250)		956	199.7	154.2	110.7	6.29
CS (250) × SM (500)		1,103	177.7	177.3	138.2	6.46
CS (500) ×SM (250)		1,097	196.2	157.8	180	7.09
CS (500) ×SM (500)		1,205	206.7	179.9	178.9	6.82

Researchers believe that beneficial intestinal bacteria have an important effect on mucosal barrier function, intestinal maturity and health, and lymphatic tissue development, and their presence is essential for intestinal barrier function (Klaver & van der Meer, 1993). Since the SM and CS extracts studied maintain activity of beneficial bacteria in the intestine and eliminate harmful ones, it is possible that via their antimicrobial and antioxidant activities, they prevent production of toxic and disturbing materials, and accordingly preserve health of intestine morphological indexes.

### 3.7 Profile of breast's fatty acids

**FIGURE 2:**
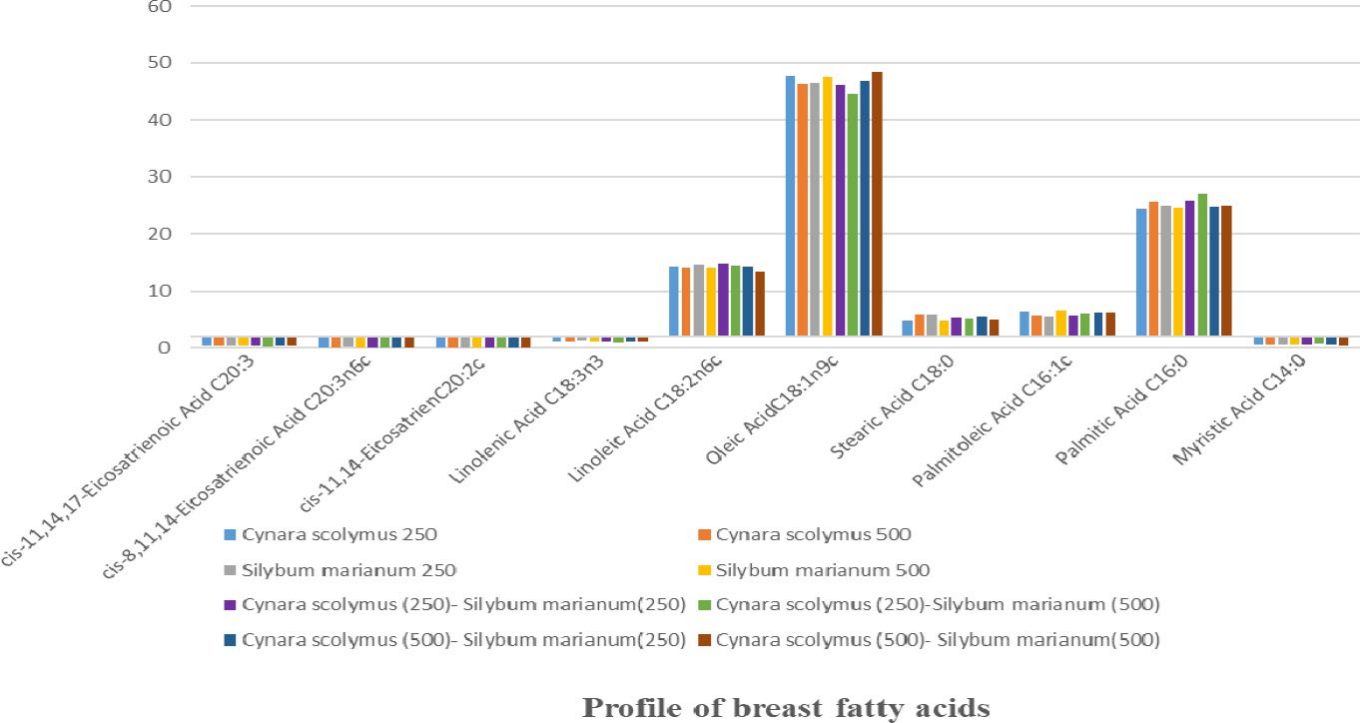
Fatty acids profile at 42 days of age of breast of Ross 308 broilers fed diets containing two levels of *Cynara scolymus* and *Silybum marianum*

The fatty acid profile of breast is presented in Table 12 and Figure 2. Concentrations of saturated fatty acids (myristic, stearic, and palmitic) increased with the increase in CS level and decreased with the increase in SM level in the diet. Saturated fatty acids with the highest frequency of cyristic and palmitic acids in breast tissue of broilers occurred when 250 mg/kg CS and 500 mg/kg SM were supplemented. However, the amount of stearic acid was highest in the chickens fed 500 mg/kg CS and 250 mg/kg SM (5.50%). The lowest amount of myristic acid (0.46%) and stearic acid (4.95%) were observed in the treatment with both supplements at 500 mg/kg. The lowest amount of palmitic acid (24.91%) was observed in the treatment of 500 mg/kg CS and 250 mg/kg SM (Table 12).

**TABLE 12:** Profile of breast fatty acids on day 42 of Ross 308 broilers fed diets containing two concentrations (250 or 500 mg/kg) of *Cynara scolymus* (CS) and *Silybum marianum* (SM)

		Myristic Acid Methyl Ester C14:0 (%)	Palmitic Acid Methyl Ester C16:0 (%)	Palmitoleic Acid Methyl Ester C16:1c (%)	Stearic Acid Methyl Ester C18:0 (%)	Oleic Acid Methyl Ester C18:1n9c (%)	Linoleic Acid Methyl Ester C18:2n6c (%)	Linolenic Acid Methyl Ester C18:3n3 (%)	cis−11,14‐Eicosadienoic Acid Methyl Ester C20:2c (%)	cis−8,11,14‐Eicosatrienoic Acid Methyl Ester C20:3n6c (%)	cis−11,14,17‐Eicosatrienoic Acid Methyl Ester C20:3 (%)
CS (mg/kg)	250	0.62	26.47	5.95	5.29	45.30	14.58	1.07	0.06	0.07	0.28
	500	0.49	24.95	6.25	5.23	47.56	13.77	1.15	0.04	0.12	0.34
SM (mg/kg)	250	0.52	25.39	6.04	5.40	46.4	14.48	1.14	0.05	0.12	0.35
	500	0.59	26.03	6.16	5.12	46.46	13.87	1.08	0.05	0.07	0.27
CS (250) ×SM (250)		0.51	25.87	5.78	5.30	46.00	14.74	1.14	0.05	0.10	0.35
CS (250) × SM (500)		0.72	27.07	6.11	5.28	44.60	14.42	1.00	0.06	0.04	0.21
CS (500) ×SM (250)		0.52	24.91	6.29	5.50	46.80	14.22	1.14	0.05	0.14	0.35
CS (500) ×SM (500)		0.46	24.99	6.21	4.95	48.32	13.31	1.15	0.03	0.10	0.33

Researchers believe that the antioxidant properties of SM are due to the flavonoid compounds present in this plant, which can affect fat metabolism (Bohm et al., 1998; Yokozawa et al., 2003). The amount of fatty acids in animal tissues is affected by dietary fatty acids concentrations. Therefore, by using supplements rich in unsaturated and essential fatty acids can produce meat that is enriched with these fatty acids. The major fatty acids in SM include palmitic, stearic, oleic, linoleic, and linolenic acids (Goli et al., 2008). Therefore, concentrations of these fatty acids should be increased in meat from broilers fed SM. Among unsaturated fatty acids, concentrations of palmitolic and oleic acids decreased with increasing concentrations of CS and increased with increasing concentration of SM in the diet. The highest concentrations of palmitolic acid were observed for the 500 mg/kg plus CS250 mg/kg SM treatment (6.29%), while the lowest concentration was observed for the 250 mg/kg dose of the both supplements (5.75%). Feeding 500 mg/kg of the two supplements resulted in the highest concentration of oleic acid (48.32%), while the 250 mg/kg CS plus 500 mg/kg SM resulted in the lowest concentration (46.60%). Increasing the levels of both extracts reduced the level of linoleic acid (13.31% versus. 14.74%) as compared to the lower doses (Table 12).

Increasing both CS and SM concentrations reduced linolenic acid, which is a member of the omega‐3 fatty acid family. The lowest concentration (1%) was observed when 250 mg/kg CS plus 500 mg/kg SM was fed, whereas the highest level (1.15%) was observed for the 500 mg/kg of both supplements. Samadi and Abbasi (2016) examined the effect of adding 15g/kg of CS leaf powder on fatty acid profile of Japanese quail carcass and reported decreased amounts of saturated fatty acids (myristic, stearic, and palmitic). However, the amount of unsaturated fatty acids was not affected by the treatment. Feeding CS leaf powder increased linolenic acid concentrations resulting in a relative improvement in the nutritional value of quail breast meat.

The three eicosatrienoic acids, cis‐11,14, cis‐8,11,14, and cis‐11,14,17, are used as a standard for biological studies, and their amount in animal tissues is very small (Huang et al., 2011; Wang et al., 2012). In the present study, the concentration of cis‐11,14‐eicosadienoic acid decreased with increasing concentration of CS and increased with increasing concentration of SM. Increasing CS increased concentrations of cis‐8,11,14‐eicosatrienoic acid and cis‐11,14, 17‐eicosatrienoic acid, while increasing SM concentrations reduced concentration of both of these fatty acids (Table 12).

## 4 CONCLUSION

Results of the present study indicate that supplementation of CS and SM in aflatoxin free diets had no effect on growth and economic performances, carcass characteristics, and immune organs, but improved antibody titer against influenza virus and sheep red blood cells. Supplementing diets with CS and SM improved blood parameters and liver enzymes. The effect of application of the extracts on profile of breast fatty acids was variable, but in general, consumption of 250 mg/kg of both supplements was effective in improving the profile of breast fatty acids.

## CONFLICT OF INTEREST

The authors declare that they have no conflict of interest.

## **AUTHOR** **CONTRIBUTIONS**

**Hossein Zaker‐Esteghamati involved in** data curation, formal analysis, funding acquisition, investigation, methodology, resources, software, and writing—original draft. **Alireza Seidavi involved in** conceptualization, data curation, funding acquisition, investigation, methodology, project administration, resources, supervision, validation, visualization, writing—original draft, and writing—review and editing. **Mehrdad Bouyeh involved in** conceptualization, data curation, formal analysis, funding acquisition, investigation, methodology, project administration, resources, software, (supervision, validation, visualization, writing—original draft, and writing—review and editing.
